# Participation in Clinical Trials Among Academic Dermatologists Affiliated With Veterans Affairs Hospitals: Survey Study

**DOI:** 10.2196/35379

**Published:** 2022-09-12

**Authors:** Torunn Sivesind, Josephine D'Angelo, Tatyana Khazova, Shahzeb Hassan, Michael Kamara, Elizabeth Wallace, Cory Dunnick, Robert Dellavalle

**Affiliations:** 1 Department of Dermatology University of Colorado Anschutz Medical Center Aurora, CO United States; 2 Department of Dermatology University of Rochester Rochester, NY United States; 3 College of Osteopathic Medicine Rocky Vista University Parker, CO United States; 4 Feinberg School of Medicine Northwestern University Chicago, IL United States; 5 School of Medicine University of Missouri Columbia, MO United States; 6 Dermatology Service, Rocky Mountain Regional Veterans Affairs Medical Center US Department of Veterans Affairs Eastern Colorado Health Care System Aurora, CO United States

**Keywords:** dermatology, dermatologists, clinical trial, COVID-19, basal cell carcinoma, psoriasis, atopic dermatitis, Veterans Affairs, carcinoma, cancer therapy, dermatologist, pandemic

## Abstract

**Background:**

Clinical trials have led to the development of new and effective therapies for many dermatologic conditions. To our knowledge, there is no published study that has quantified and described the degree of involvement in clinical trials among academic dermatologists and their university affiliates.

**Objective:**

The purpose of this study was to characterize the involvement of academic dermatology departments in clinical trials research.

**Methods:**

An online survey was sent to 211 Veterans Affairs (VA)–employed dermatologists. It comprised 20 questions related to the number of clinical trials, support staff dedicated to clinical research, skin diseases studied, and the effect of the COVID-19 pandemic on conducting clinical research. Three rounds of survey invitations were sent over a 3-month period (March to May 2021). Data from all survey responses were reviewed for quantitative and descriptive analyses of the key outcome measures.

**Results:**

A total of 48 dermatologists completed the survey and provided their university affiliations and details of involvement in clinical trials research. Over half of participants (n=25, 58.1%) with a university affiliate reported that their affiliated dermatology department had a dedicated clinical trials unit. Basal cell carcinoma was the most frequently studied skin condition (n=9, 18.8%), followed by atopic dermatitis and psoriasis (n=4, 8.3% each); 66.7% of participants reported no current clinical trials participation. Of those conducting clinical trials, 87% (n=18) noted that COVID-19 was a barrier to conducting trials, with 52.2% (n=11) citing disrupted or decreased trials due to the pandemic.

**Conclusions:**

Although many dermatologists with university affiliations reported having a dedicated clinical trials unit at their institution, a majority of those surveyed reported not taking part in any active trials. Overall, the diseases investigated in academic clinical trials appear to follow national trends, though some of the top dermatological diseases are underrepresented in clinical trials research. A key limitation of our study was the low response rate (~23%) and that the survey responses from the sample of VA-based dermatologists may not be generalizable to all academic dermatology departments in the United States. The effect of the COVID-19 pandemic appeared to play a significant role in disrupting active trials.

## Introduction

Skin conditions are a common and burdensome health problem in the United States, with 1 in 3 people affected at a given time [1]. These conditions are associated with negative emotional effects and a reduced quality of life, which contribute to increased direct and indirect medical costs [1], and underscore the importance of establishing efficacious treatment options. Recent advances in our understanding of dermatologic diseases have enabled the development of cutting-edge drugs and procedures. At the forefront of these developments are clinical trials, with common skin diseases such as psoriasis and atopic dermatitis well represented in clinical trials research [2]

A clinical trial, as defined by the International Committee of Medical Journal Editors, is research that prospectively assigns human participants to intervention and concurrent comparison groups to study the cause-and-effect relationship between a medical intervention and a health outcome [3]. Clinical trials test treatment efficacy, advance our knowledge of medical procedures, and may offer access to more beneficial drugs compared to standard therapeutic options.

Although clinical trials are essential for the advancement of contemporary medicine and the field of dermatology, clinical trials research among Veterans Affairs (VA)–affiliated academic dermatology departments and their associated institutions reportedly varies by type and degree of participation [1]. To our knowledge, there have been no studies characterizing the degree to which academic dermatology departments are involved in clinical trials, nor any that address which dermatologic conditions are most frequently studied and which are currently underrepresented in clinical trials. We sought to characterize the involvement of academic dermatology departments in clinical trials research with respect to the institutional support provided for clinical trials, the conditions studied, availability of research support, and barriers to conducting clinical research.

## Methods

### Overview

An online survey was created via SurveyMonkey (Momentive Inc) (see Multimedia Appendix 1 for complete survey questions and response summary) and was piloted among local VA dermatologists, who provided feedback and contributed to the final survey design. The final survey consisted of 20 questions asking for information related to the number of clinical trials, support staff dedicated to clinical research, skin diseases studied, and the effect of the COVID-19 pandemic on conducting clinical research. VA-based dermatologists were identified by a listserve and invited to participate in an online survey-based assessment. Three rounds of survey invitations were sent over a 3-month period (March to May 2021), with 211 potential participants contacted via email and invited to participate.

Data from all survey responses were reviewed for quantitative and descriptive analyses of the key outcome measures. Two reviewers (MK and TK) independently tabulated survey responses, and a third reviewer (TS) confirmed the results.

### Ethical Considerations

The study was granted an exemption by the Colorado Multi-Institutional Review Board and was approved by the VA Eastern Colorado Health Care System Subcommittee on Research Safety.

## Results

A total of 48 VA dermatologists (48/211, 22.7%) completed the survey. Responses are summarized in Multimedia Appendix 1. All dermatologists who were surveyed reported having active VA appointments and currently seeing patients, with 16 (33.3%) reporting participation in clinical trials research. More than half of all respondents (n=31, 66%) reported not currently studying any dermatologic conditions, although 38.7% (12/31) of these reported prior (n=6) or planned (n=7) clinical trials research (includes 1 participant who reported both prior and planned research). The majority (n=43, 89.6%) reported an affiliation with the dermatology department of a university; 25 (58.1%) reported their university’s dermatology department had a dedicated clinical trials unit.

Among the dermatologic conditions currently being investigated in clinical trials, basal cell carcinoma was the most frequently studied (participants: n=9, 18.8%; institutions: 26.7%), followed by atopic dermatitis and psoriasis (participants: tied at n=4, 8.3%; institutions: 10.0%). Of survey participants, 39.6% (n=19) reported no involvement in clinical trials. Lack of time (n=29, 60.4%) and lack of resources (n=30, 62.5%) were cited most frequently as barriers to involvement in clinical trials research. For those who reported active participation in clinical trials, most were involved in 1 or 2 trials (n=6, 12.5% each; total: n=12, 25%). Among those who reported participation in clinical trials research, 52.2% noted an interruption or decline in clinical trials research secondary to the COVID-19 pandemic; 13% cited lack of time due to increased clinical and administrative duties, and another 8.7% were unable to recruit adequate numbers of staff or new patients. The remaining 13% stated that COVID-19 had a limited effect on their ability to conduct clinical trials. Comments describing the impact of COVID-19 on clinical research appear in Textbox 1.

**Additional comments regarding the effect of COVID-19 on clinical trials research. VA: Veterans Affairs.**

1. “Both University and VA were designated COVID hospitals. The U. is live again; the VA is evaluating studies on a case by case basis. None reopened yet.”
2. “Cancelled one trial - very limited support from the VA - will be leaving the VA soon due to lack of support for research”
3. “Clinical trial placed on hold and could not enroll. When we could enroll, patient no longer qualified”
4. “Continued to do studies with established patients. Couldn't start new trials or enroll new patients for a while”
5. “Currently not actively engaged in projects; however, have been limited in the number of patients able to be seen, and projects, in general, have been placed on hold.”
6. “Decreased activity severely”
7. “Delays”
8. “Fewer clinical trials”
9. ”For the VA in particular, key staff were limited in terms of availability and responsiveness. For the university affiliate, there was nearly a month delay in IRB review/approvals related to holiday closure and longer time off for staff (related to COVID surge)”
10. “Had to pause one clinical trial, yet two are epidemiologic studies for which we are struggling to find statistical support”
11. “Hard to keep up with clinical duties”
12. “Have not been able to do research”
13. “I have had to focus on clinical and administrative work (vs science) almost exclusively since COVID.”
14. “Lab and trials were shut down for many months and now only partially active”
15. “Limited recruitment for a period of time”
16. “Lack of access to patients, and money/resources”
17. “Limited effect”
18. “COVID-19 has not affected my trials.”
19. “Patient numbers at our institution are severely limited. Would make doing research very challenging, since we can't get all our veterans with active problems seen in a timely fashion”
20. “Slowed it”
21. “Trials put on hold. Trial coordinator working virtually.”
22. “VA staff who participate are focused on COVID response and don't prioritize participation in research”
23. “Only doing clinic now”

## Discussion

### Principal Findings

Our study revealed that basal cell carcinoma was the most frequently studied skin problem among VA dermatologists, likely correlating to the high incidence of skin cancer in the VA population [4]. Psoriasis and atopic dermatitis were the second most commonly studied conditions. This is in line with other recent work [5] that suggests an ongoing trend of psoriasis and atopic dermatitis being among the most researched skin conditions. Assessment of the clinical trials database maintained by the US National Library of Medicine [6] as of July 22, 2021, similarly reflects the prominence of psoriasis and atopic dermatitis among dermatologic trials, demonstrating that, for the conditions reported in our survey, psoriasis accounts for the greatest number of active trials (n=727) nationwide, followed by atopic dermatitis (n=450 active trials) and basal cell carcinoma (n=176 active trials).

A comparison of the conditions reported in this study to epidemiological data (1990-2017) from the Global Burden of Disease study [7] and its disability-adjusted life-year estimates for the most burdensome dermatological conditions in the United States [8] shows that several top skin diseases (including acne, alopecia areata, contact dermatitis, urticaria, and viral and fungal skin diseases) were not investigated by any of the survey respondents in our study. However, this could be due to the small sample size and low response rate failing to capture all diseases being studied by academic dermatology departments in clinical trials.

In addition to gauging involvement in clinical trials among VA dermatologists and their associated academic institutions, this study sought to understand the impact of COVID-19 on participation in clinical trials. Though survey participants reported lack of time and resources as the greatest barriers overall to pursuing clinical trials research, COVID-19 was noted to pose new challenges such as disruptions and delays in trials, lack of access to patients and resources, and increased clinical obligations. Other studies have likewise found the pandemic poses many obstacles for clinical researchers and study participants, including, but not limited to, site closures, mandatory self-isolation, travel restrictions, interrupted delivery of investigational products, infection of staff or study participants, significant delays in the enrollment of subjects, and difficulty in study monitoring [9,10].

### Limitations

Limitations of this study include a low survey response rate (~23%) and inclusion of only VA-affiliated dermatologists. We estimate that the listserve used to distribute surveys may have missed approximately 46 VA-affiliated dermatologists who would have been eligible to participate; inclusion of these dermatologists would increase the denominator and further decrease the survey response rate.

### Conclusion

Our study suggests that academic dermatology departments are conducting clinical trials in line with current national trends, with atopic dermatitis and psoriasis at the forefront of clinical trial efforts. It remains a challenge to balance patient care and clinical research missions within academic dermatology departments. Additionally, securing adequate support in the form of qualified study personnel and financial resources support to conduct high-quality research can be a barrier to maintaining clinical trials units, especially during the COVID-19 pandemic. Further work should be done to survey academic dermatology departments directly and to compare the results with published trials on ClinicalTrials.gov. Use of the ClinicalTrials.gov database could also include surveying the full list of dermatology-related clinical trials site locations to see which of these are affiliated with academic institutions. Such studies would provide a more robust assessment of investment in clinical trials research among teaching hospitals and dermatology faculties.

## Appendix

Multimedia Appendix 1 Survey with response summary.

## Abbreviations

VA: Veterans Affairs
