# Supplementary material for: Participation in Clinical Trials Among Academic Dermatologists Affiliated With Veterans Affairs Hospitals: Survey Study
Source: JMIR Dermatol. 2022 Sep 12;5(3):e35379. doi: 10.2196/35379 (PMC9511003; doi:10.2196/35379)
Supplement: Multimedia Appendix 1 [file derma_v5i3e35379_app1.docx]

**Multimedia Appendix 1. Survey with response summary.**

| **1. What is your role at the VA?**  __Staff Physician (Dermatology)  __Staff Physician (specialty other than Dermatology)  __Midlevel Provider (NP, PA, etc.)  __Other (please specify) | 48 responses (missing data=0):  48 (100%)  0 (0%)  0 (0%)  0 (0%) |
| --- | --- |
| **2. Do you have an active VA appointment?**  __Yes  __No  __Other (please specify) | 48 responses (missing data = 0)  48 (100%)  0 (0%)  0 (0%) |
| **3. Do you see patients currently?**  __Yes  __No  __Other (please specify) | 48 responses (missing data = 0)  48 (100%)  0 (0%)  0 (0%) |
| **4. Do you participate in clinical trials research?**  __Yes, currently  __Previously  __Planning to in the future  __No  __Other (please specify) | 48 responses (missing data = 0)  16 (33.3%)  5 (10.4%)  6 (12.5%)  19 (39.6%)  1*(2.1%)    *(previously and planning to in the future) |
| **5. For which dermatologic conditions are you currently conducting clinical trials?**  __Acne  __Alopecia  __Atopic dermatitis  __Basal cell carcinoma  __Epidermolysis bullosa  __Hidradenitis  __Lupus  __Melanoma  __Pemphigoid  __Pemphigus  __Psoriasis  __Pyoderma gangrenosum  __Squamous cell carcinoma  __Urticaria  __Vitiligo  __Other (please specify) | 48 responses (missing data = 0) _________________  0 (0%)  0 (0%)  4 (8.3%)  9 (18.8%)  1 (2.1%)  0 (0%)  1 (2.1%)  1 (2.1%)  1 (2.1%)  1 (2.1%)  4 (8.3%)  1 (2.1%)  3 (6.3%)  0 (0%)  0 (0%)  11 (22.9%)  *(diabetic wounds, skin cancer, tinea pedis, onychomycosis, allergic contact dermatitis, cutaneous T-cell lymphoma, dermatomyositis, Merkel cell carcinoma, teledermatology, non-invasive imaging, wound healing, all N=1 response per)    **NB:** multiple selections were allowed per respondent; percentages are out of total respondents |
| **6. How many active trials are you participating in?**  __1  __2  __3  __4  __5  __>5  __Other (please specify) | 48 responses (missing data = 0)  6 (12.5%)  6 (12.5%)  0 (0%)  1 (2.1%)  1 (2.1%)  1 (2.1%)  33 (68.8%)*    *(none currently: N=16; N/A: N=16; other unspecified: N=1) |
| **7. Are you involved in cooperative studies through the VA?**  __Yes  __No  __Other (please specify) | 48 responses (missing data = 0) _________________  4 (8.3%)  39 (81.3%)  5 (10.4%)*    *(N/A) |
| **8. Do you conduct clinical research through VA clinical merit awards?**  __Yes  __No  __Other (please specify) | 48 responses (missing data = 0) _________________  4 (8.3%)  40 (83.3%)  4 (8.3%)*    *(N/A) |
| **9. Do you conduct industry-sponsored research?**  __Yes  __No  __Other (please specify) | 48 responses (missing data = 0)  8 (16.7%)  35 (72.9%)  5 (10.4%)*    *(N/A) |
| **10. Do you conduct basic science research?**  __Yes  __No  __Other (please specify) | 48 responses (missing data = 0)  8 (16.7%)  36 (75.0%)  4 (8.3%)*  *(N/A) |
| **11. Are you affiliated with the dermatology department of a university?**  __Yes  __No  __Other (please specify) | 48 responses (missing data = 0) _________________  43 (89.6%)  5 (10.4%)  0 (0%)* |
| **12. With which university (or primary university, if multiple) are you affiliated?** | 48 responses (missing data = 0) _________________  Columbia (N=1)  Dartmouth (N=1)  Emory (N=2)  Indiana University (N=2)  Loyola (N=1)  Medical University of South Carolina (N=1)  None currently (N=1)  N/A (N=7)  Oregon Health Sciences University (N=1)  Stanford (N=1)  State University, unspecified (N=1)  SUNY Downstate (N=1)  UC Davis (N=2)  UC Irvine (N=1)  UCSF (N=2)  University of Colorado (N=2)  University of Connecticut (N=2)  University of Kentucky (N=1)  University of Miami (N=1)  University of Missouri (N=2)  University of New Mexico (N=1)  University of Iowa (N=1)  University of Pennsylvania (N=1)  University of Southern California (N=1)  University of South Florida (N=1)  University of Texas, Dell (N=1)  University of Texas, San Antonio (N=2)  University of Utah (N=1)  University of Washington (N=1)  Vanderbilt (N=1)  Virginia Commonwealth University (N=3)  Wayne State University (N=1) |
| **13. Does your university’s dermatology department have a dedicated clinical trials unit?**  __Yes  __No  __Other (please specify) | 48 responses (missing data = 0) _________________  25 (52.1%)  16 (33.3%)  7 (14.6%)*    *(N/A: N=6; other: N=1) |
| **14. How many clinical trials coordinators are employed?**  __0  __1  __2  __3  __4  __5 or more  __Uncertain/don’t know | 48 responses (missing data = 0)  3 (6.3%)  8 (16.7%)  5 (10.4%)  1 (2.1%)  1 (2.1%)  2 (4.2%)  20 (41.7%) |
| **15. Does the university’s dermatology department provide financial support for the clinical trials unit? (e.g., pays the salary of the clinical trials coordinator outside of income generated by the trial)**  __Yes  __No  __Uncertain/don’t know  __Other comments (please specify) | 48 responses (missing data = 0)      8 (16.7%)  13 (27.1%)  22 (45.8%)  5 (10.4%)*    *(N/A) |
| **16. Have you encountered any barriers to conducting clinical research?**  __Yes  __No  __Other (please specify) | 48 responses (missing data = 0) _________________  26 (54.2%)  11 (22.9%)  12 (25.0%)*    *(N/A: N=6, other: N=6) |
| **17. What barriers limit your clinical trials participation?**  __Lack of time  __Lack of resources  __Other (please specify) | 48 responses (missing data = 0)  29 (60.4%)  30 (62.5%)  5 (10.4%)*    *(Lack of knowledge: N=3; hiring difficulty: N=1; lack of support: N=1) |
| **18. Do you have access to a clinical research fellow (at the VA or via university association) for project assistance?**  __Yes  __No  __Other (please specify) | 48 responses (missing data = 0)    13 (27.1%)  31 (64.6%)  4 (8.3%)*    *(N/A: N=2, other: N=2) |
| **19. Is the research fellow able to participate in industry sponsored research?**  __Yes  __No  __Other (please specify) | 48 responses (missing data = 0) _________________  10 (20.8%)  12 (25.0%)  26 (54.2%)*    *(N/A: N=15; uncertain: N=9; not as part of a fellowship: N=2) |
| **20. Please describe how COVID-19 affected your ability to conduct or maintain clinical trials research.**  **Note: all percentages rounded** | 48 responses (missing data = 0)  **See **Table 1** for verbatim comments” |
